# Centralized care model for hereditary angioedema overcomes geographical barriers

**DOI:** 10.3389/fimmu.2024.1413547

**Published:** 2024-07-15

**Authors:** Ashley Holmes, Cindy Srinivasan, Jack Borle, Heather Blain, Bruce Ritchie, Adil Adatia

**Affiliations:** ^1^ Faculty of Science, University of Alberta, Edmonton, AB, Canada; ^2^ Faculty of Medicine, University of Alberta, Edmonton, AB, Canada; ^3^ Alberta Precision Laboratories, Edmonton, AB, Canada; ^4^ Division of Hematology, Department of Medicine, University of Alberta, Edmonton, AB, Canada; ^5^ Division of Pulmonary Medicine, Department of Medicine, University of Alberta, Edmonton, AB, Canada

**Keywords:** hereditary angioedema, *SERPING1*, rural medicine, kallikrein, C1 inhibitor, immunodeficiency

## Abstract

Hereditary angioedema due to C1 inhibitor deficiency (HAE) is a rare inborn error of immunity that presents with episodic swelling. Management is multifaceted and includes on-demand treatment of swelling episodes, short-term prophylaxis to prevent swelling episodes from procedures, and long-term prophylaxis (LTP) to prevent angioedema on an ongoing basis. All approved on-demand therapies are parenteral, necessitating patient training for home administration, particularly intravenous C1 inhibitor. These complexities can result in care gaps for rural HAE patients. We conducted a cross-sectional study at our Angioedema Center of Reference and Excellence to assess the care provided to urban and rural patients. The proportion of patients receiving LTP, proportion of patients diagnosed as children, and disease control measured using the Angioedema Control Test (AECT) were collected. Logistic and Poisson regression models adjusted for age and sex were used to compare the two groups. The proportion using LTP was similar at 62% and 61% in urban and rural patients, respectively (odds ratio [OR] 1.01 (CI 95% 0.34-2.99)). Among urban patients, 52% were diagnosed as children compared to 60% among rural residents (1.43 (0.37-5.56)). The mean (IQR) AECT score was 14.0 (8.5-15.5) in urban patients and 13.0 (10.0-14.0) in rural patients (Poisson β -0.001 (-0.23-0.23). These data indicate that rural patients received similar high-quality care. We attribute these findings to the centralized care model employed in which HAE patients in the region are seen at a single comprehensive care clinic.

## Introduction

Hereditary angioedema due to C1 inhibitor deficiency (HAE) is a genetic disease characterized by recurrent swelling of the cutaneous and submucosal tissues (1). It is caused by mutations in *SPERING1*, which lead to reduced or dysfunctional C1 inhibitor (C1-inh) (2). C1-inh is the primary regulator of the kinin-kallikrein pathway and acts through the inhibition of plasma kallikrein and Factor XIIa. The absence of effective C1-inh, which normally inhibits plasma kallikrein from overproducing bradykinin through the proteolysis of high-molecular-weight kininogen, results in angioedema as bradykinin increases vascular permeability (3).

HAE care is multifaceted and complex, leading to significant challenges for rural patients (4–6). Local physicians must recognize the condition despite the low disease prevalence of around 1 in 50,000 persons and arrange testing or specialist consultation (1). Definitive diagnosis requires testing of C1-inh function, which is often only performed at tertiary care centers (7). Once diagnosed, patients need education on the appropriate self-administration of on-demand therapy, including self-injection training since all approved on-demand therapies are parenteral. This is of particular importance for those who use intravenous C1-inh for on-demand treatment as patients must be competent to obtain intravenous access. For those who reside in small communities, arrangements must be made to ensure they have access to on-demand treatments in the local emergency department (ED) as well. Ongoing care additionally involves organizing short-term prophylaxis with intravenous C1-inh to prevent angioedema episodes triggered by invasive procedures (*e.g.*, dental surgery). In many cases, long-term prophylaxis (LTP) to prevent attacks with regular treatment is needed to reduce the disease burden and achieve an acceptable quality of life (1). Given the autosomal dominant inheritance of the disease, genetic counseling and screening of first-degree relatives is also indicated (8).

A recent physician survey regarding the difficulties faced by rural HAE patients found that ~12% of HAE patients resided in a remote community and highlighted delays in diagnosis and cost of therapy as key issues (6). Riedl et al. conducted a systematic literature review on HAE care in rural patients and identified several important challenges, including barriers to diagnosis, access to specialist care, and availability of on-demand treatments for home and ED use (5). There is, however, a paucity of data to inform policies and clinical programs designed to overcome these geographical barriers.

## Methods

We conducted a cross-sectional study to evaluate the quality of care provided to urban and rural patients with HAE seen at the Edmonton Angioedema Center of Reference and Excellence (9). This clinic provides centralized HAE care for patients residing in Edmonton, Alberta, Canada (population of 1.1 million) and rural patients living in northern parts of Alberta and British Columbia, and western parts of Nunavut. The approximate geographic size of the catchment area is 1.9 million km^2^.

The study was approved by the University of Alberta Biomedical Research Ethics Board. Individual consent was waived given that the study was designed as an audit of clinical records. Included patients had HAE type 1 or 2, were ≥18 years of age, and were assessed at least once at the Edmonton ACARE between January 1 and March 31, 2023. Demographic data, postal code, use of LTP, age at diagnosis, and angioedema control (based on the angioedema control test score [AECT] (10)) were extracted from the most recent clinical records. Patients were classified as rural using the Statistics Canada preferred definition of those residing in census subdivisions with <10,000 inhabitants who are outside city commuting zones (11, 12). Logistic regression was used to calculate the odds ratios for LTP use and diagnosis before age 18 in rural *versus* urban patients. Poisson regression was used to compare the mean AECT score between the two groups. All models were adjusted for age and sex.

## Results

**Table 1** shows the demographic data of the included patients. Approximately 38% of patients were classified as living in a rural area. The average straight-line distance to the clinic for rural patients was 504.6 km whereas the average straight-line distance for urban patients was nearly 6-fold less at 86.7 km. Four patients resided in highly remote areas that were accessible only by aircraft.

**Table 1:** Demographics of urban and rural HAE patients seen at Edmonton ACARE.

	All patients (n=60)	Rural (n=23)	Urban (n=37)
Age (years), mean (SD)	44 (17)	43 (19)	44 (16)
Sex (female), n (%)	42 (70)	14 (61)	28 (76)
Intubation, n (%)‡	6 (11)	1 (5)	5 (15)
Hospitalization, n (%)‡	14 (27)	4 (21)	10 (31)
Throat attack, n (%)‡	34 (61)	14 (70)	20 (56)
Abdominal attack, n (%)‡	49 (87)	18 (90)	31 (86)
Mean straight-line distance to Edmonton ACARE, km (SD)	246.9 (421.9)	504.6 (575.7)	86.7 (138.8)
Mean road distance to Edmonton ACARE, km (SD) ^†^	176.0(221.3)	315.1(252.4)	104.6 (151.5)
AECT score, median (IQR)	13.0 (9.0-15.0)	13.0 (10.0-14.0)	14.0 (8.5-15.5)

^†^Locations that were too remote to have road access were excluded from road distance calculations.

‡Proportions of patients with any previous history of event including those which occurred prior to diagnosis of HAE.

Most patients were receiving LTP, similar to other centers in developed countries (13). All patients on LTP were receiving subcutaneous/intravenous pd-C1, lanadelumab, berotralstat, or an investigational product; no patients were treated with attenuated androgens or tranexamic acid, consistent with international guideline recommendations (1).

There was no difference between the quality of rural and urban HAE care based all metrics assessed (**Table 2**). LTP usage was comparable at 62% and 61% in urban and rural patients, respectively (odds ratio [OR] 1.01 (CI 95% 0.34-2.99)). Of urban residents, 52% were diagnosed as children compared to 60% as adults (1.43 (0.37-5.56)). The mean (IQR) AECT score comparable at 14.0 (8.5-15.5) in urban patients and 13.0 (10.0-14.0) in rural patients (Poisson β -0.001 (-0.23-0.23), and indicated adequate control (>10) in both groups.

**Table 2:** Comparison of long-term prophylaxis use, diagnosis before adulthood, and AECT score between patients living in urban and rural areas.

Dichotomous variables	Urban areas	Rural areas	OR (95% CI)
Long-term prophylaxis ^a^	23 (62%)	14 (61%)	1.01 (0.34-2.99)
Age at diagnosis <18 ^a^	16 (52%)	12 (60%)	1.43 (0.37-5.56)
**Count variable**			**β (95% CI)**
AECT score ^b^	14 (8.5-15.5)	13 (10-14)	-0.001 (-0.23-0.23)

Data are shown as ^a^ odds ratio (OR) with 95% confidence intervals (CIs) obtained from logistic regressions for dichotomous variables and ^b^ regression coefficient (β) with 95% CI for count variable obtained from Poisson regression. All models were adjusted for age and sex.

## Discussion

This is the largest primary study comparing the care provided to HAE patients who reside in rural and urban areas. More than 1/3 of patients resided in a rural community, many of whom lived several hours away by vehicle. Nonetheless, use of LTP, angioedema control, and frequency of diagnosis in childhood were comparable between urban and rural patients. We ascribe the absence of inequity in these measures to the centralized care model employed for HAE. Rather than distributing care amongst smaller local clinics, concentrating patients within a tertiary care center has many advantages. It allows the provision of comprehensive care with specialist physicians and allied health team members, implementation of best practices in telehealth care delivery, creation a single point of contact for rural providers who require advice, and creation of processes to navigate insurance procedures for medication coverage.

The implications of these findings are broad. A recently published US physician survey found that most rural patients were cared for in local clinics. Some of the geographical challenges faced by patients could thus potentially be addressed by leveraging tertiary care centers and telehealth (6). Studies have reported broad benefits telemedicine in clinical immunology and allergy clinics since its adoption increased during the COVID-19 pandemic (14, 15). Additionally, globally many patients with HAE live in developing nations in the Asia Pacific region, a large proportion of whom reside in rural areas, and up to 99% of affected patients are thought to be undiagnosed in some countries (16, 17). The use of digital health technologies coordinated through specialized HAE centers to provide expert advice to rural healthcare providers and directly to patients using smartphones could help address these care gaps as has been described for other diseases (18).

These findings must be considered with the context of the Alberta health care system. Canadian Blood Services provides access to subcutaneous pd-C1 LTP (Haegarda/Berinert 2000) to all patients irrespective of private insurance coverage. Lanadelumab and berotralstat were also available for most patients and there is significant local experience in using these drugs as first line LTP therapies (19, 20). Those residing in extremely remote regions can be provided with transportation services funded by territorial health systems to attend appointments. Physician reimbursement of telehealth is also comparable to in-person visits in most cases.

This study has limitations. Data were collected from a single center located in a highly developed country with universal health coverage and thus may not be generalizable to other settings. Whether a patient resided in a rural area was determined using their current postal code. We could thus not account for whether they previously resided in a different area. The use of census subdivisions with <10,000 residents as a metric for rural residency may not fully capture the challenges experienced by HAE patients in all cases.

In conclusion, the creation of centralized HAE clinics employing a comprehensive care model can help rural patients overcome barriers to quality HAE care and achieve disease outcomes comparable to those in urban patients. Implementing such a care model using networks of centralized clinics in underserved countries may help address global inequities in HAE outcomes.

## Data availability statement

The raw data supporting the conclusions of this article will be made available by the authors, without undue reservation.

## Ethics statement

The studies involving humans were approved by University of Alberta Research Ethics Board. The studies were conducted in accordance with the local legislation and institutional requirements. The ethics committee/institutional review board waived the requirement of written informed consent for participation from the participants or the participants’ legal guardians/next of kin because This study was conducted as a clinical audit.

## Author contributions

AH: Writing – review & editing, Writing – original draft. CS: Writing – review & editing. JB: Writing – review & editing. HB: Writing – review & editing. BR: Writing – review & editing, Writing – original draft. AA: Writing – review & editing, Writing – original draft.
